# Supplementary material for: Causal Relationship between Aging and Anorexia Nervosa: A White-Matter-Microstructure-Mediated Mendelian Randomization Analysis
Source: Biomedicines. 2024 Aug 16;12(8):1874. doi: 10.3390/biomedicines12081874 (PMC11351342; doi:10.3390/biomedicines12081874)
Supplement: Supplementary file 1 [file biomedicines-12-01874-s001.zip › Supplementary Figure/Figure S4/Singe SNP plot of MR analysis for TL to ubm-b-1640.pdf]

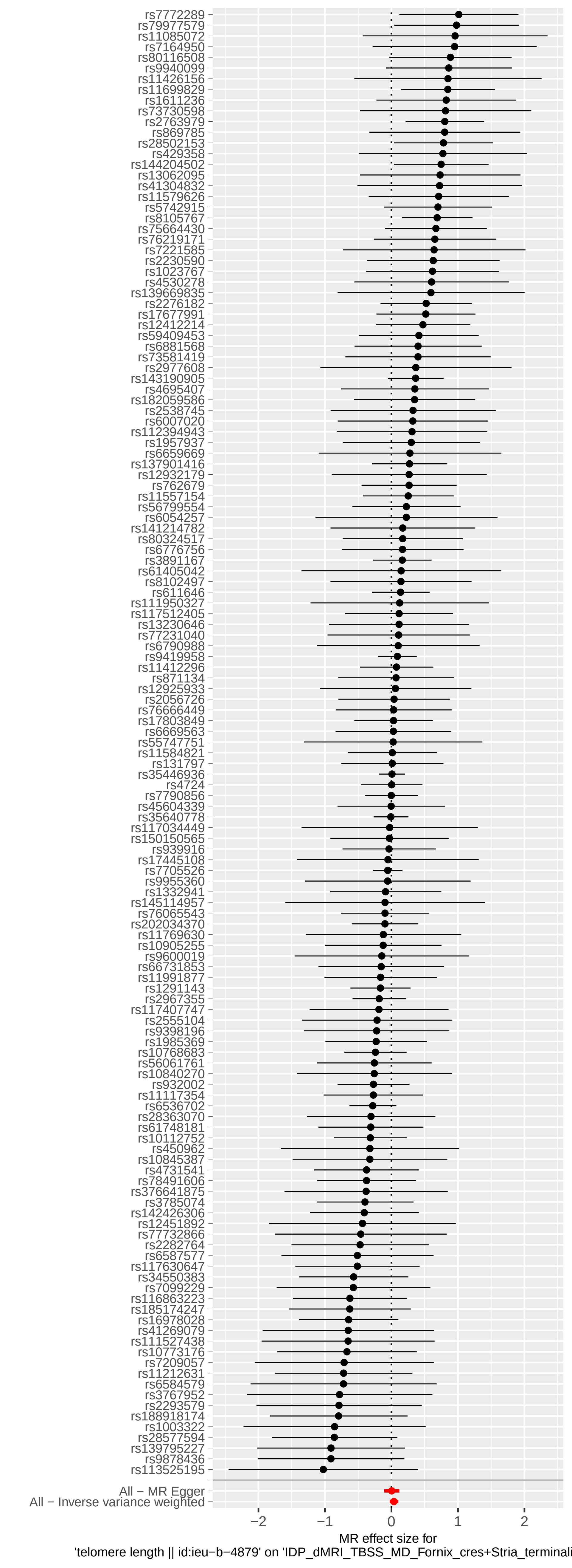

rs7772289
rs79977579
rs11085072
rs7164950
rs80116508
rs9940099
rs11426156
rs11699829
rs1611236
rs73730598
rs2763979
rs869785
rs28502153
rs429358
rs144204502
rs13062095
rs41304832
rs11579626
rs5742915
rs8105767
rs75664430
rs76219171
rs7221585
rs2230590
rs1023767
rs4530278
rs139669835
rs2276182
rs17677991
rs12412214
rs59409453
rs6881568
rs73581419
rs2977608
rs143190905
rs4695407
rs182059586
rs2538745
rs6007020
rs112394943
rs1957937
rs6659669
rs137901416
rs12932179
rs762679
rs11557154
rs56799554
rs6054257
rs141214782
rs80324517
rs6776756
rs3891167
rs61405042
rs8102497
rs611646
rs111950327
rs117512405
rs13230646
rs77231040
rs6790988
rs9419958
rs11412296
rs871134
rs12925933
rs2056726
rs76666449
rs17803849
rs6669563
rs55747751
rs11584821
rs131797
rs35446936
rs4724
rs7790856
rs45604339
rs35640778
rs117034449
rs150150565
rs939916
rs17445108
rs7705526
rs9955360
rs1332941
rs145114957
rs76065543
rs202034370
rs11769630
rs10905255
rs9600019
rs66731853
rs11991877
rs1291143
rs2967355
rs117407747
rs2555104
rs9398196
rs1985369
rs10768683
rs56061761
rs10840270
rs932002
rs11117354
rs6536702
rs28363070
rs61748181
rs10112752
rs450962
rs10845387
rs4731541
rs78491606
rs376641875
rs3785074
rs142426306
rs12451892
rs77732866
rs2282764
rs6587577
rs117630647
rs34550383
rs7099229
rs116863223
rs185174247
rs16978028
rs41269079
rs111527438
rs10773176
rs7209057
rs11212631
rs6584579
rs3767952
rs2293579
rs188918174
rs1003322
rs28577594
rs139795227
rs9878436
rs113525195
All − MR Egger
All − Inverse variance weighted
−2
−1
0
1
2
MR effect size for
'telomere length || id:ieu−b−4879' on 'IDP_dMRI_TBSS_MD_Fornix_cres+Stria_terminali
